# Machine Learning Predicts Biogeochemistry from Microbial Community Structure in a Complex Model System

**DOI:** 10.1128/spectrum.01909-21

**Published:** 2022-02-09

**Authors:** Avishek Dutta, Thomas Goldman, Jeffrey Keating, Ellen Burke, Nicole Williamson, Reinhard Dirmeier, Jeff S. Bowman

**Affiliations:** a Integrative Oceanography Division, Scripps Institution of Oceanography, UC San Diego, La Jolla, California, USA; b BP Biosciences Center, San Diego, California, USA; c Center for Microbiome Innovation, UC San Diego, La Jolla, California, USA; University of Michigan-Ann Arbor

**Keywords:** biogeochemical state, machine learning, random forest, sulfidogenesis potential, microbial community analysis

## Abstract

Microbial community structure is influenced by the environment and in turn exerts control on many environmental parameters. We applied this concept in a bioreactor study to test whether microbial community structure contains information sufficient to predict the concentration of H_2_S as the product of sulfate reduction. Microbial sulfate reduction is a major source of H_2_S in many industrial and environmental systems and is often influenced by the existing physicochemical conditions. Production of H_2_S in industrial systems leads to occupational hazards and adversely affects the quality of products. A long-term (148 days) experiment was conducted in upflow bioreactors to mimic sulfidogenesis, followed by inhibition with nitrate salts and a resumption of H_2_S generation when inhibition was released. We determined microbial community structure in 731 samples across 20 bioreactors using 16S rRNA gene sequencing and applied a random forest algorithm to successfully predict different phases of sulfidogenesis and mitigation (accuracy = 93.17%) and sessile and effluent microbial communities (accuracy = 100%). Similarly derived regression models that also included cell abundances were able to predict H_2_S concentration with remarkably high fidelity (R^2^ > 0.82). Metabolic profiles based on microbial community structure were also found to be reliable predictors for H_2_S concentration (R^2^ = 0.78). These results suggest that microbial community structure contains information sufficient to predict sulfidogenesis in a closed system, with anticipated applications to microbially driven processes in open environments.

**IMPORTANCE** Microbial communities control many biogeochemical processes. Many of these processes are impractical or expensive to measure directly. Because the taxonomic structure of the microbial community is indicative of its function, it encodes information that can be used to predict biogeochemistry. Here, we demonstrate how a machine learning technique can be used to predict sulfidogenesis, a key biogeochemical process in a model system. A distinction of this research was the ability to predict H_2_S production in a bioreactor from the effluent bacterial community structure without direct observations of the sessile community or other environmental conditions. This study establishes the ability to use machine learning approaches in predicting sulfide concentrations in a closed system, which can be further developed as a valuable tool for predicting biogeochemical processes in open environments. As machine learning algorithms continue to improve, we anticipate increased applications of microbial community structure to predict key environmental and industrial processes.

## INTRODUCTION

Microorganisms are important contributors to biogeochemical cycles and also play an important role in determining elemental fluxes in a system. Environmental conditions, substrate, and nutrient availability are often the key players in determining the microbial community structure and function, which in turn control the biogeochemical transformations and fluxes in a system. These transformations can be either beneficial or detrimental to specific members of the microbial community, leading to a shift in community composition. The bilateral link between biogeochemistry and microbial community composition suggests that either state should inform the other, e.g., that a specific microbial community suggests a particular biogeochemical state (1).

The sulfur cycle is one of the most complex microbially mediated biogeochemical cycles because sulfur has a broad range of oxidation states from −2 (completely reduced) to +6 (completely oxidized) and can undergo both biotic and abiotic transformation (2). Often, these sulfur transformations are coupled to the carbon and nitrogen cycle. This makes carbon and nitrogen compounds important determinants of sulfur transformations in a system. One such process is dissimilatory sulfate reduction by sulfate-reducing bacteria (SRB); SRB use sulfate as a terminal electron acceptor for the degradation of organic compounds resulting in the production of H_2_S (2). H_2_S production adversely affects different industrial processes and can pose health and safety concerns (3–5). This makes it imperative to understand the sulfidogenesis potential of a system to pursue proper mitigations.

Interestingly, the introduction of nitrate in the system inhibits the production of sulfidogenesis and is often used in oil recovery and wastewater treatment processes to suppress sulfidogenesis (3, 4, 6–10). The addition of nitrate salts stimulates the growth of nitrate-reducing bacteria (NRBs) (3, 11). The heterotrophic NRBs outcompete SRBs by drawing down the pool of volatile fatty acids and other electron donors, while chemolithotrophic nitrate-reducing sulfur-oxidizing bacteria (NR-SOBs) have the additional benefit of reducing the H_2_S concentration (3, 11, 12). This study evaluates a machine learning (ML)-based method to predict biogeochemical state and H_2_S concentrations of a complex system from microbial community structure, where interplay among different sulfur, carbon, and nitrogen compounds creates a dynamic system.

Considering the broad applications of ML techniques in other fields, relatively few studies have applied ML techniques to problems in microbial ecology. Changes in environmental conditions shape the microbial community in a unique manner that affects the emergent geochemical properties of the system. These properties are represented not only by a specific functional guild (e.g., the SRBs) but also by other populations of microbes that are directly or indirectly influenced by changing conditions. Hence, the microbial community in aggregate can be considered a meta-indicator of conditions in an ecosystem. One recent study demonstrated the application of machine learning models in classifying healthy and *Fusarium* wilt-diseased soils based on microbial community data (13). Another study demonstrated the use of a deep learning approach in predicting microbial interactions from self-organized spatiotemporal patterns (14). Though several studies have applied ML-based approaches in the field of microbiology (15), the application of ML for predicting microbial community function is limited. Bowman et al. (16) used self-organizing maps to predict bacterial production based on bacterial community structure along the western Antarctic Peninsula. Thompson et al. (17) used neural networks and random forest (RF) approaches to predict dissolved organic carbon based on soil microbiome in a plant litter decomposition experiment. These studies indicate that ML approaches can be used as a tool for understanding high-dimensional microbial data sets. Among different ML algorithms, the RF model has been proven to be one of the most efficient ML approaches with high classification accuracy for exploring various 16S rRNA data sets to predict habitats, hosts, health status, and community functions (13, 17, 18). Moreover, the nonparametric nature of RF models and their ability to assess the contributions of specific features (19) make them suitable for many problems in microbial ecology.

Here, we applied RF to predict microbial community state and a key geochemical parameter (H_2_S concentration) in a lab-scale microcosm experiment representing a complex natural system. Forty-nine distinct RF models were constructed from random subsamples of 731 observations from a 148-day-long *ex situ* experiment, which mimicked different phases of sulfidogenesis and mitigation. We explored the robustness of RF models in predicting sulfidogenesis and mitigation phases, the sulfidogenesis potential of a microbial community, and microbial community source (sessile or planktonic) by using different sets of microbial taxa as independent variables. We anticipate that this approach can be generalized to predict many biogeochemical processes in different systems, even if the observed microbial community is only indirectly related to the process. Because a microbial assemblage typically consists of many thousands of members, it is sensitive to or causative of many environmental parameters. This sensitivity allows it to serve as a hypersensitive indicator of environmental change. Given adequate training data, we anticipate that many different environmental parameters can be predicted from community structure data in a given microbial system.

## RESULTS

### Shift in microbial community structure across different phases of sulfidogenesis and mitigation.

**FIG 1:**
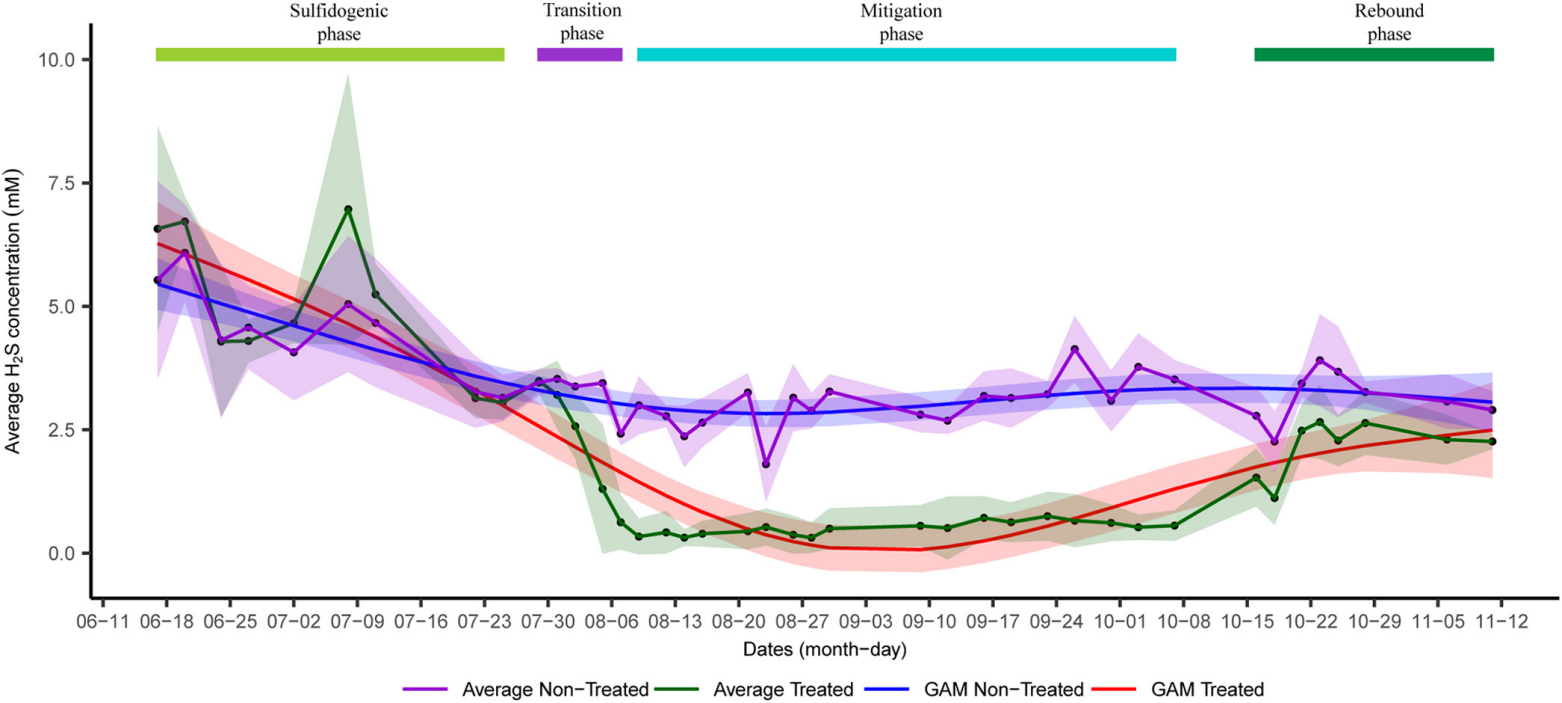
Shift in average sulfide concentration across different phases in treated and nontreated columns. Shaded regions for generalized additive models (GAM) indicate ±2 standard error, whereas shaded regions for average plot indicate standard deviation.

**FIG 2:**
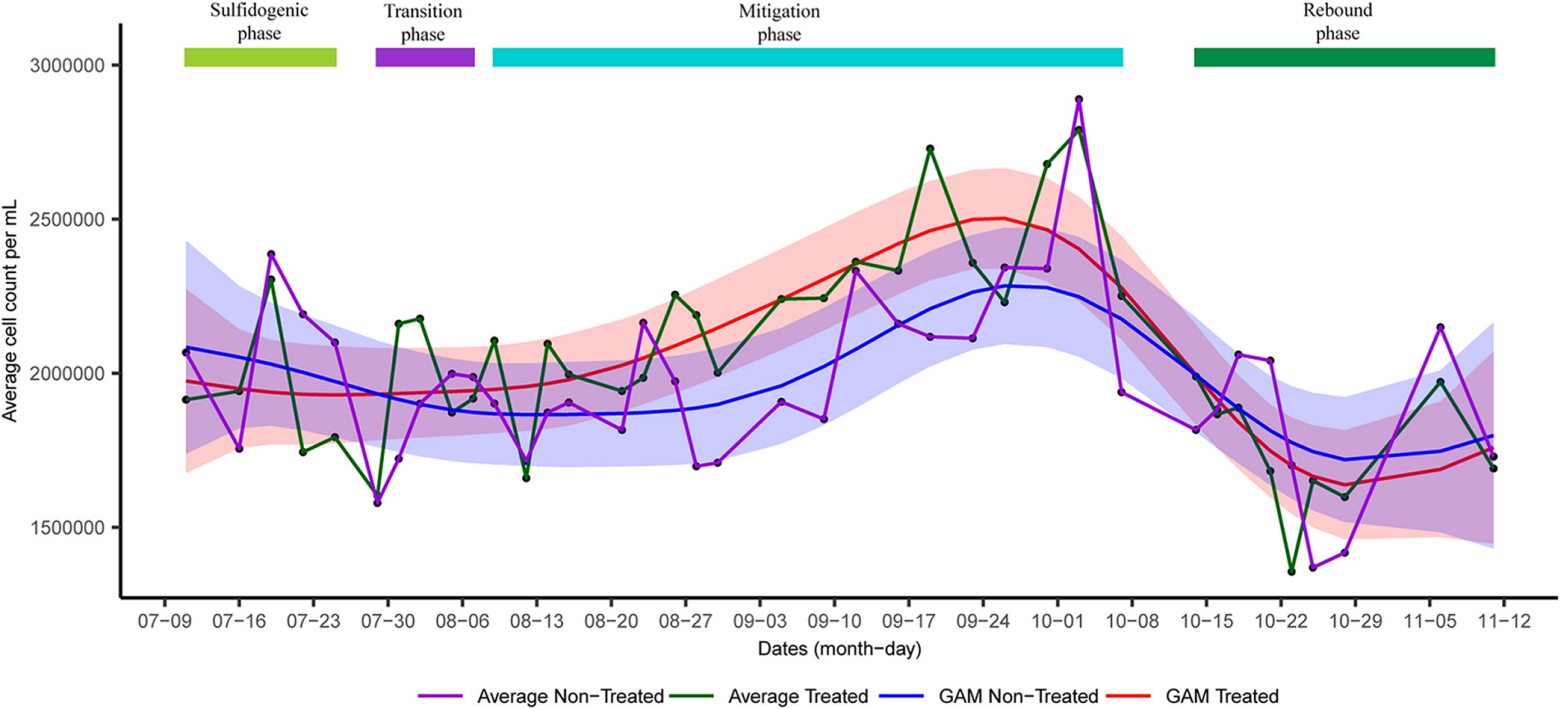
Shift in average cell abundances across different phases in treated and nontreated columns. Shaded regions for generalized additive models (GAM) indicate ±2 standard error.

Twenty upflow bioreactors were used to understand the shift in microbial diversity across sulfidogenesis and mitigation phases. Out of 20 bioreactors, nitrate salts were added in 10 columns (referred to as treated columns) to suppress sulfidogenesis, whereas the other 10 were used as controls where no nitrate salts were added (referred to as nontreated columns). Three main phases were observed in the treated columns, *viz*, sulfidogenic, mitigation, and rebound sulfidogenesis (referred to as rebound in the manuscript). The mitigation phase was achieved when nitrate salts were added to the system to suppress sulfidogenesis, whereas the rebound phase was achieved when nitrate treatment was stopped (Fig. 1). Generalized additive models (GAMs) based on average H_2_S concentration across different time points in the treated columns indicated a decrease in average H_2_S concentration when nitrate salts were added and an increase in average H_2_S concentration when nitrate treatment was stopped. Comparison of GAMs based on average H_2_S concentrations in treated and nontreated columns indicated that nitrate treatment led to suppression of sulfidogenesis. A transition to the mitigation phase (referred to as transition in this study) between sulfidogenesis and mitigation was determined where the H_2_S concentration was >1 mM even after the nitrate treatment. Average cell abundances also shifted across different phases (Fig. 2). GAMs based on average cell abundances across treated columns suggested an increase in cell abundance during nitrate addition, followed by a decrease in cell abundance when nitrate treatment was stopped.

**FIG 3:**
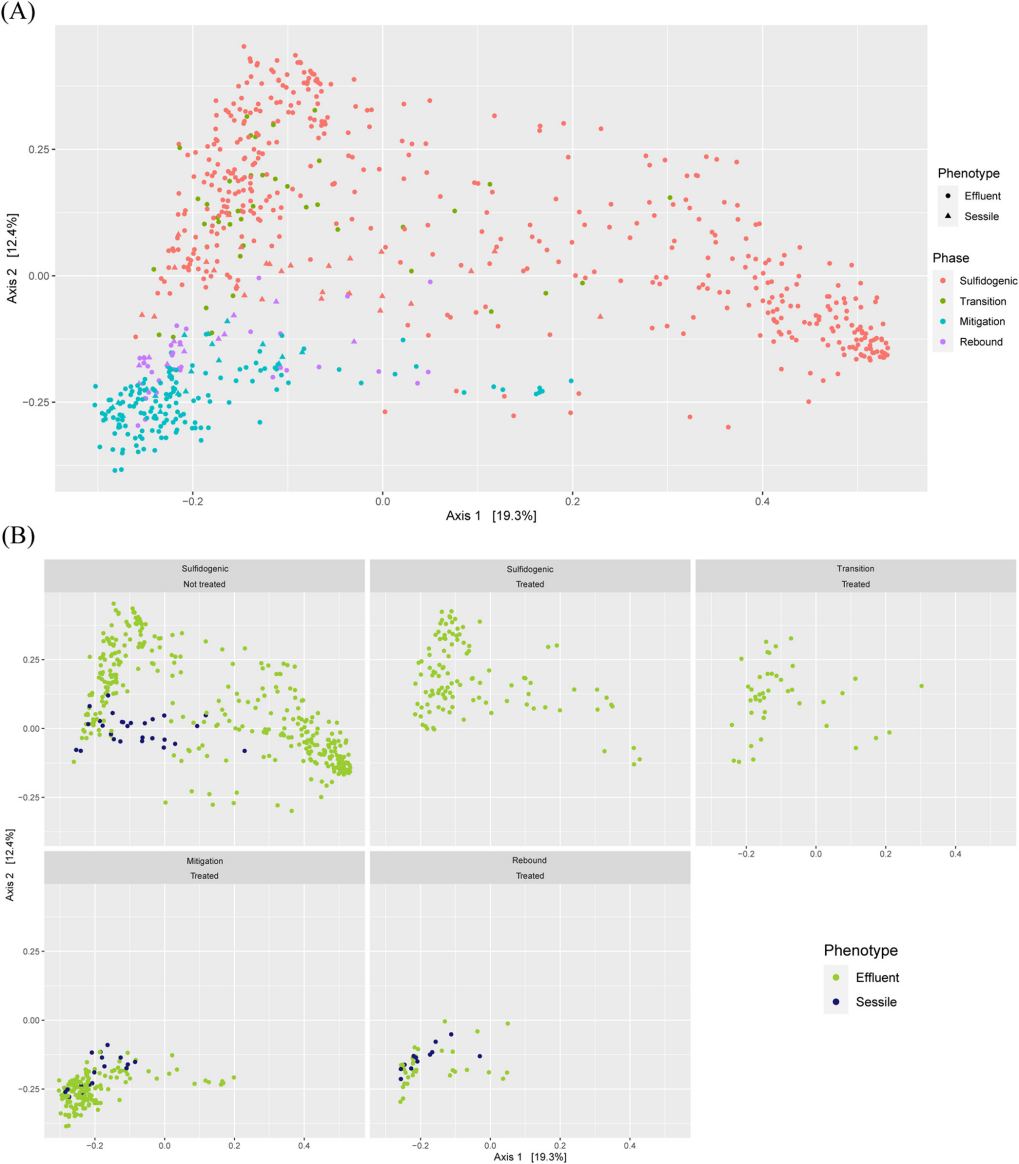
Principal coordinates analysis (PCoA) of Bray-Curtis dissimilatory based on the relative abundance of all unique sequences. (A) PCoA plot showing samples from all the phases. (B) Facet plots of the same PCoA emphasizing sessile and effluent communities across different phases in treated and nontreated columns.

We applied principal coordinates analysis (PCoA) to see if the experimental results met our initial assumption that mitigation induces dynamic shifts in microbial community structure. PCoA showed that the microbial community shifted across different phases (Fig. 3), with distinct clusters observed for sulfidogenesis and mitigation phases. Samples from the sulfidogenic phase on the PCoA plot were found to be widely distributed. The samples from the transition phase clustered with the samples from the sulfidogenic phase, whereas the rebound phase samples grouped closer to the mitigation phase samples, which indicates that microbial community shifts are gradual and not instantaneous in this system. The PCoA plots also indicated that sessile and effluent microbial populations were similar for a particular phase.

### Microbial community composition as a determinant of phases and a predictor for H_2_S concentration.

**FIG 4:**
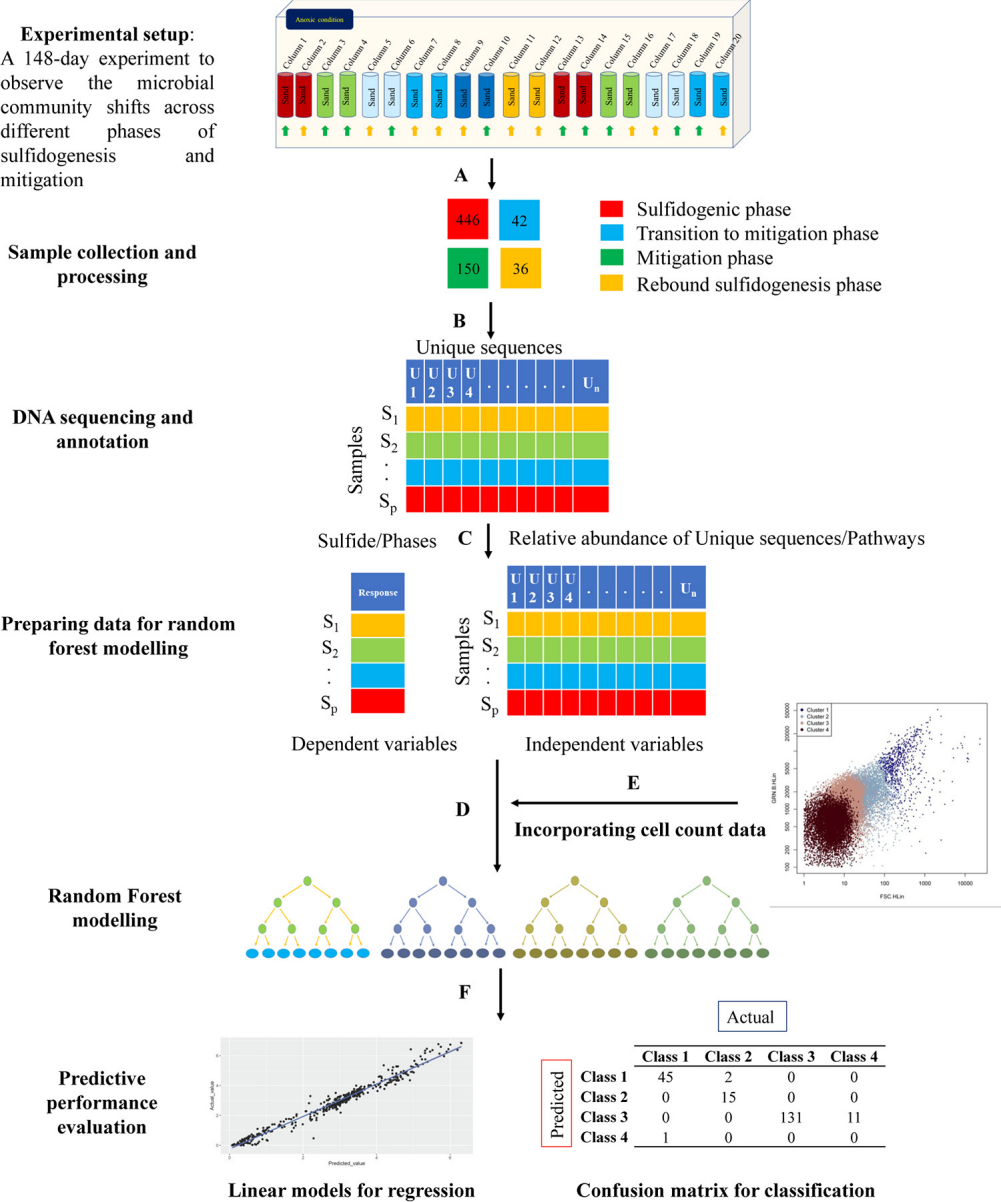
Overall pipeline for random forest prediction of H_2_S production. The green arrows depict treated columns, whereas the yellow arrows depict nontreated columns in the experimental setup. (A) Sample collection and DNA extraction from effluent samples from different phases. (B) High-throughput 16S rRNA gene sequencing followed by paprica analysis. (C) Preparing data set for random forest modeling. (D) Classification and regression-based random forest modeling depending on the dependent variables. (E) Integrating cell count data to calculate the absolute abundance of unique sequences and using them as independent variables for predicting phases and H_2_S concentration. (F) Evaluating the predictive performance of the data using linear models (for regression models) and confusion matrix (for classification).

An approach based on the RF algorithm was developed to predict sulfidogenesis potential in upflow bioreactors based on microbial community structure across different phases of sulfidogenesis and mitigation (Fig. 4). Briefly, the bioreactors were sampled at different time points to yield 674 effluent samples (see Table S1 in the supplemental material). Microbial community structure was described from 16S rRNA gene sequencing using phylogenetic placement with the paprica pipeline. The relative abundances of unique bacterial sequences across different samples were used as independent variables, whereas phases and H_2_S concentration measured during the sample collection time points were used as dependent variables for prediction with RF models. To further enhance and compare the RF models, cell abundances of different microbial taxa determined by flow cytometry were used as independent variables. To generalize and increase the applicability of the models, predicted metabolic structure (in the form of pathway abundances) for each sample as inferred from paprica based on microbial community structure was also used as an independent variable.

Classification-based RF models were used for the prediction of phases, whereas regression-based RF models were used for the prediction of H_2_S concentration. For classification-based models predicting phases, we used 674 observations of relative abundance of unique bacterial sequences and predicted metabolic pathways (termed relative abundance models), whereas 535 observations were used for the absolute models based on relative abundance multiplied by cell count (termed absolute abundance models) (Table 1). For regression-based models, the data set was filtered based on H_2_S data availability, outliers, and other inconsistencies. A total of 593 observations were used for relative abundance data sets (both unique and pathway abundances), whereas 475 observations were used for the absolute abundance data set for predicting H_2_S concentrations using regression-based models. Since out-of-bag (OOB) error (for classification models) and percentage variance explained (for regression models) statistics provided in RF show the goodness of model fit, but not necessarily predictive performance (20, 21), 30% of the observations in all the regression and classification models were randomly withheld (the validation data set) and were used to perform more precise model validation. We refer to the remaining 70% of the observations as the training data set. For regression models, the variations in H_2_S concentrations in the validation data set and the training data set were kept similar to minimize the chance of underfitting the model.

**TABLE 1:** Details of classification-based random forest models showing accuracies for training and validation^*a*^

Random forest model	Data used	No. of observations	Prediction	NIV	No. of trees	*mtry*	OOB	Accuracy training	Accuracy validation
CM 1	Relative abundance of unique sequences	674	Phases	12,713	300	112	8.96%	1	0.9317
CM 2	Absolute abundance of unique sequences	535	Phases	10,887	300	104	9.92%	1	0.9211
CM 3	Relative abundance of pathways	674	Phases	809	300	28	8.74%	1	0.922
CM 4	Relative abundance of unique sequences	114	Sessile-effluent	5,300	300	72	0.00%	1	1

a NIV, number independent variables; OOB, out-of-bag estimate of error rate.

Since H_2_S concentration varied across phases, it was essential to understand the accuracies of the RF models in predicting phases from the microbial community data set before the models were used for the prediction of H_2_S concentration. RF models for predicting phases were highly accurate (Table 1). OOB estimate of error for all the phase-predicting models (CM1, CM2, CM3) was lower than 10%. A confusion matrix was further used to assess the accuracies of the RF models in predicting phases based on training and validation data set (Table S2 to S4). All the classification models had 100% accuracy in predicting the phases from the training data set. Accuracies for predicting phases from the validation data set for all the phase prediction models were similar (93.17%, 92.11%, and 92.2% for CM 1, CM 2, and CM 3, respectively). It was interesting to note that the pathway-based model (CM 3) performed equally well compared to the relative (CM 1) and absolute (CM 2) abundance models, even when CM 3 had a number of independent variables notably lower than that of CM 1 and CM 2. The OOB estimate of error for CM 3 was also found to be the lowest among the three phase-predicting models (Table 1). For these three phase-predicting models, all the phases except for the transition phase were predicted with high accuracy (Table S2, S3, and S4). Most of the transition phase observations were predicted as sulfidogenesis phase by these three phase-predicting models.

**FIG 5:**
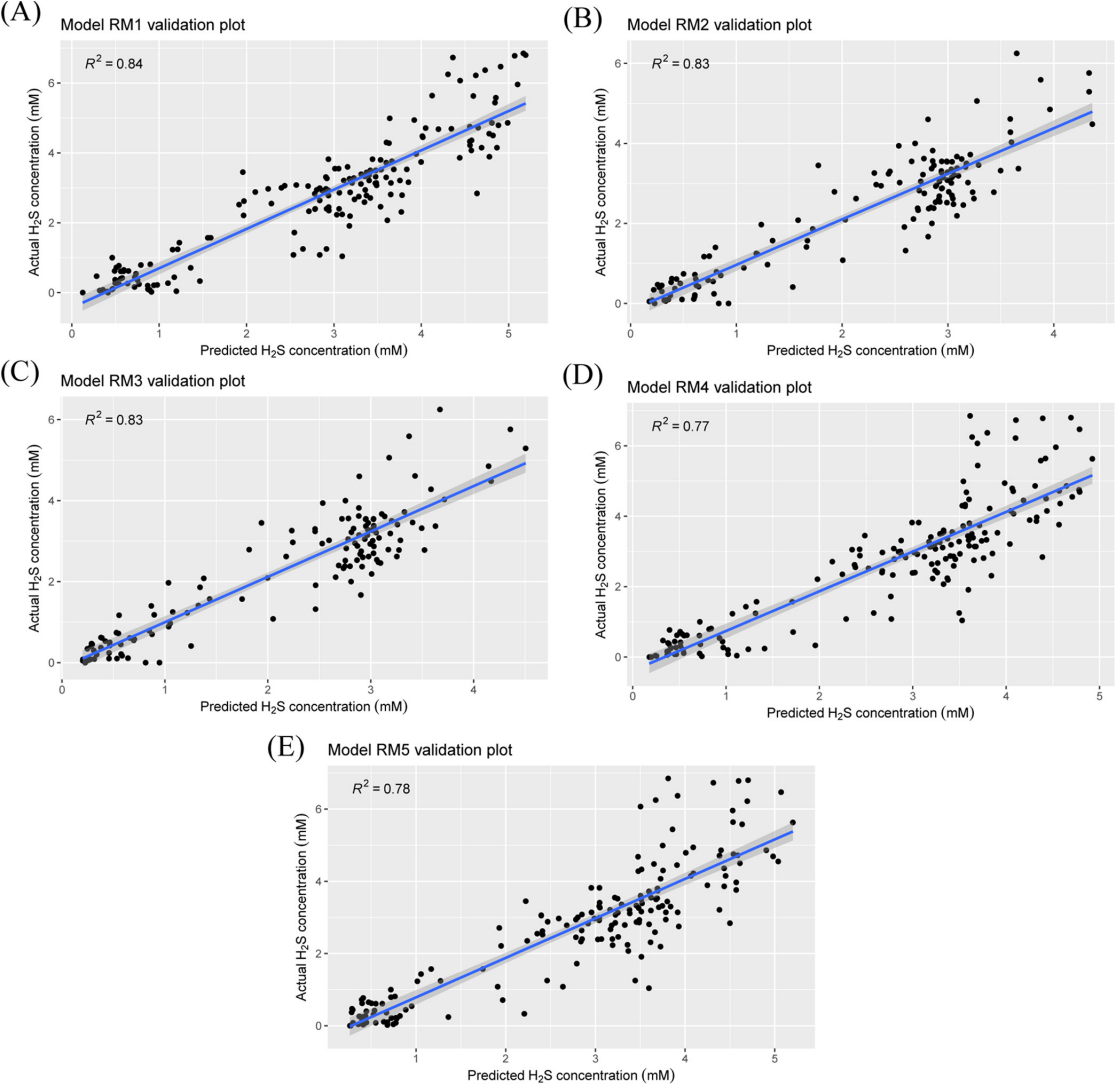
H_2_S concentration prediction with random forest regression models. (A) Scatterplot of predicted versus actual H_2_S concentration from validation set (*n* = 174) of RM 1 (based on relative percentage abundance of bacterial unique sequence). (B) Scatterplot of predicted versus actual H_2_S concentration from validation set (*n* = 135) of RM 2 (based on the absolute abundance of bacterial unique sequence). (C) Scatterplot of predicted versus actual H_2_S concentration from validation set (*n* = 135) of RM 3 (based on relative percentage abundance of bacterial unique sequence). (D) Scatterplot of predicted versus actual H_2_S concentration from validation set (*n* = 174) of RM 4 (based on relative percentage abundance of pathways). (E) Scatterplot of predicted versus actual H_2_S concentration from validation set (*n* = 174) of RM 5 (based on relative percentage abundance of feature-selected pathways).

Five models were built to assess the applicability of RF in predicting H_2_S concentration (Table 2, Fig. 5). Though goodness of fit and predictive performance varied among the different models, all the models predicted H_2_S concentration with high accuracy. The percentage variance explained (which can also be considered pseudo-R^2^) was lower for RM 1 (relative abundance model) than for RM 2 (absolute abundance model). Linear regression of actual and predicted H_2_S was used to determine the accuracies (from R^2^) and the predictive performance of the models (Fig. 5). Both RM 1 and RM 2 had high accuracies in predicting H_2_S concentration from the validation data set (R^2^ = 0.8378 for RM 1 and R^2^ = 0.8273 for RM 2). For comparison, a subset of the RM 1 data set was taken to match the absolute abundance data set of RM 2 in terms of observations. This subset was used for model RM 3, and it was found that the predictive performance of RM 2 (R^2^ = 0.8273) was marginally better than that of RM 3 (R^2^ = 0.8258).

**TABLE 2:** Details of regression-based random forest models for predicting sulfide concentrations^*a*^

Random forest models	Data used	No. of observations	No. of trees	*mtry*	NIV	MSR	PVE	R^2^ training	RSE training	R^2^ validation	RSE validation
RM 1	Relative abundance of unique sequences	593	300	3,879	11,637	0.5737	79.11	0.9736	0.27	0.8378	0.715
RM 2	Absolute abundance of unique sequences	475	300	3,352	10,058	0.3766	80.99	0.9763	0.2174	0.8273	0.6124
RM 3	Relative abundance of unique sequences	475	300	3,352	10,058	0.3666	81.49	0.9768	0.2152	0.8258	0.615
RM 4	Relative abundance of pathways	593	300	266	799	0.6768	75.36	0.9706	0.2847	0.7692	0.853
RM 5	Relative abundance of pathways (VSURF)	593	300	11	33	0.5813	78.83	0.9716	0.2799	0.7776	0.8372

a NIV, number of independent variables; MSR, mean of squared residuals; PVE, percentage variance explained; RSE, residual standard error.

**FIG 6:**
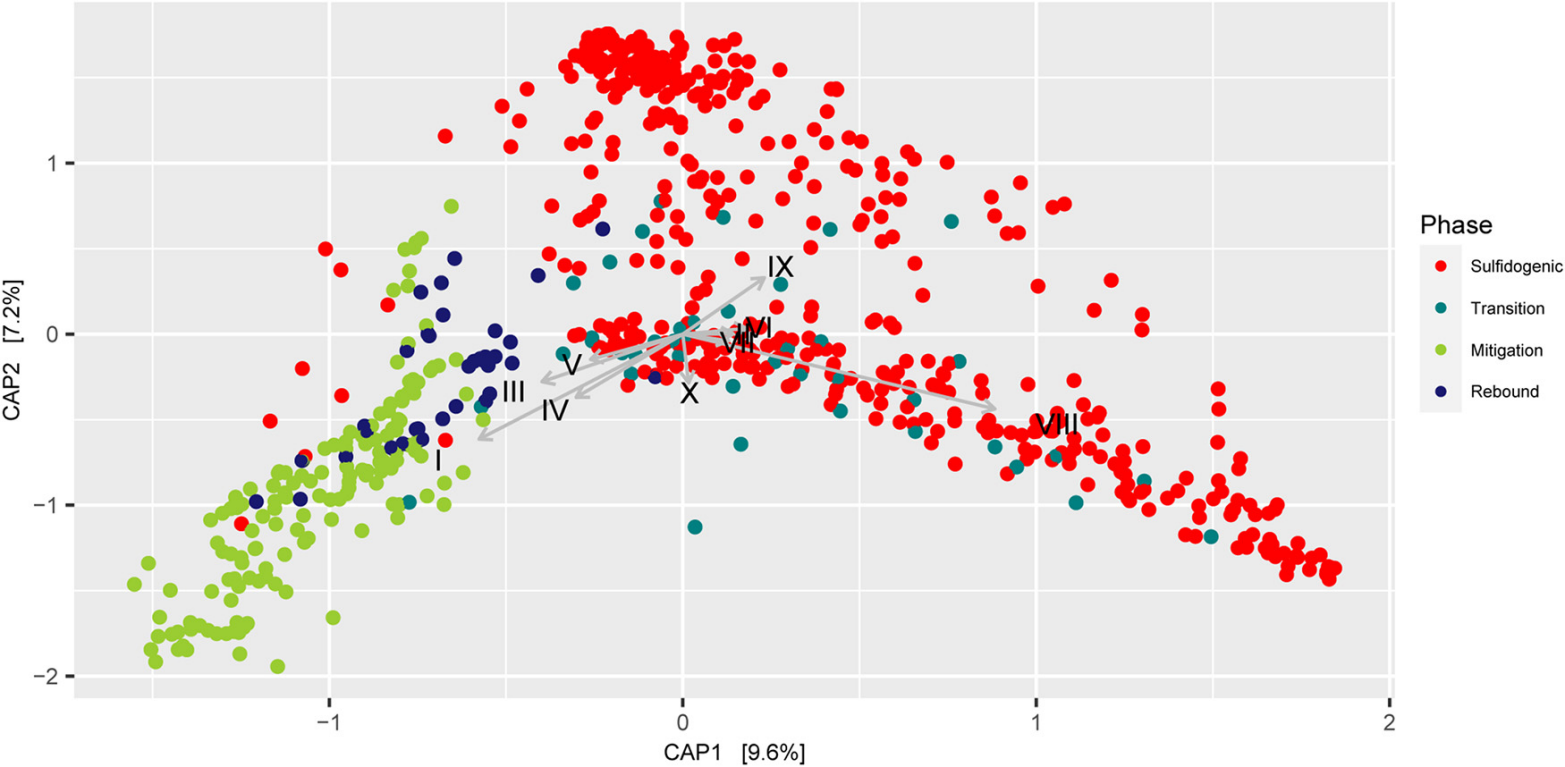
Canonical Analysis of Principal coordinates (CAP) of Bray-Curtis dissimilarity based on the relative abundance of unique sequences across all the effluent samples and constraining the 10 most important taxa, which are determinants for H_2_S concentration. I, *Denitrovibrio*, II, *Phaeobacter*, III, *Cohaesibacter*, IV, *Halarcobacter*, V, *Maritalea*, VI, *Amylibacter*, VII, *Formosa*, VIII, *Shimia*, IX, *Parcubacteria*_genera_incertae_sedis, X, *Neptunitalea*. Detailed taxonomic affiliations are mentioned in Table 3.

Assessment of feature contribution to the RF models was explored to understand the taxa that best predict the H_2_S concentration in this system. RM 1 was found to have the best predictive performance (R^2^ = 0.8378 for the validation data set) and was explored further to understand the most deterministic taxa. The 10 most deterministic taxa (based on unique sequence taxonomy) were selected based on the percentage increase in mean squared error (Table 3). Canonical Analysis of Principal coordinates (CAP) was done to determine the differential presence of the 10 most deterministic taxa across different phases (Fig. 6). CAP constraining the top 10 taxa displayed distinct clusters for samples from mitigation and sulfidogenic phases. The samples from the sulfidogenic phase were more widely distributed than the mitigation phase samples. The taxonomic affiliations of the unique sequences as determined by paprica were confirmed using the RDP classification of paprica edges (ROPE) pipeline (https://github.com/avishekdutta14/ROPE) and used for CAP analyses. ROPE uses the RDP classifier (22) trained with RDP 16S rRNA training set for taxonomic affiliation of the unique sequences obtained from paprica. Four (*Denitrovibrio*, *Cohaesibacter*, *Halarcobacter*, and *Maritalea*) of the top 10 taxa were relatively more abundant in the mitigation phase samples, whereas 5 (*Phaeobacter*, *Amylibacter*, *Formosa*, *Shimia*, and *Candidatus* Parcubacteria genera *incertae sedis*) of the top 10 taxa were relatively more abundant in the sulfidogenic phase samples. Though the majority of the top 10 taxa were found to be higher in the sulfidogenic phase, it was interesting to note that out of the top 5 most deterministic taxa, 4 of the taxa were found to be higher in the mitigation phase, whereas only 1 taxon was found to be higher in the sulfidogenic phase.

**TABLE 3:** Top 10 important taxa (based on unique sequence taxonomy) critical for the prediction of H_2_S concentration^*a*^

CCG/CEG	ROPE taxonomy	%IncMSE
*Denitrovibrio acetiphilus* DSM 12809	*Denitrovibrio*_genus_0.99	15.87
*Phaeobacter inhibens*	*Phaeobacter*_genus_0.97	15.58
*Hartmannibacter diazotrophicus*	*Cohaesibacter*_genus_1.0	14.64
*Campylobacteraceae*	*Halarcobacter*_genus_0.58	13.36
*Maritalea myrionectae*	*Maritalea*_genus_0.9	10.35
*Octadecabacter*	*Amylibacter*_genus_0.97	9.80
*Formosa* sp. Hel3_A1_48	*Formosa*_genus_0.93	9.12
*Tateyamaria omphalii*	*Shimia*_genus_0.73	9.11
Not detected	*Parcubacteria*_genera_incertae_sedis_genus_0.51	7.27
*Euzebyella marina*	*Neptunitalea*_genus_0.22	6.82

a CCG, closest completed genome; CEG, closest estimated genome; %IncMSE, percent increase in mean squared error; ROPE-based taxonomic affiliation is in the following format, taxon name_taxonomic rank_confidence of taxonomic affiliation.

Relative percentage abundances of pathways were also used as independent variables (RM 4) in H_2_S-predicting RF models to improve the generalizability. A large reduction in the number of independent variables (from 11,637 unique sequences to 799 predicted pathways) was observed in the pathway-based model (RM 4) compared to that in relative (RM 1) and absolute (RM 2) abundance-based models. The prediction accuracy of RM 4 (R^2^ = 0.7692 for validation data set) was found to be lower than that of RM 1 and RM 2. To further improve the pathway-based model and to achieve a parsimonious model, feature selection was conducted using the VSURF package (23). Feature selection from the pathway data set not only reduced the number of independent variables from 799 (RM 4) to 33 (RM 5) but also increased the percentage variance explained (PVE) and prediction accuracy and at the same time decreased the mean of squared residual (MSR) of the pathway-based model (Table 2).

### Cross-validation for determining the robustness of random forest models for predicting H_2_S concentrations.

The robustness of the RF models and their generalizability to a pseudoindependent data set were tested with a cross-validation approach. In this approach, samples from a particular column were used as a validation set, whereas the observations from the remaining 19 columns were used as a training set. Since the microbial communities experienced different dynamics in different columns, this analysis was made to analyze RF model robustness and to predict the shift in H_2_S concentration of a particular column which was excluded from the training data. Twenty different RF models were constructed and evaluated based on the relative abundance of unique sequences (Table 4, Fig. S1). Percent variance explained was high for all 20 models (mean = 82.143, standard deviation [SD] = 0.828). Assessment of predictive performance from the validation data set yielded variable results. R^2^ values were high for the columns where nitrate treatment was applied (mean R^2^ = 0.843, SD = 0.089), whereas R^2^ values were much lower for the control columns where nitrate treatment was not applied (mean R^2^ = 0.452, SD = 0.184). Percent residual standard error (RSE %) was calculated based on the average H_2_S concentration in each column to compare all 20 models. RSE % for the treated columns (mean RSE % = 34.732, SD = 9.831) were significantly higher (*t *=* *3.796, *P = *0.002) than those for the nontreated columns (mean RSE % = 21.899, SD = 4.202).

**TABLE 4:** Details of cross-validation models for predicting sulfide concentrations based on relative abundance of unique sequences^*a*^

Random forest model	Validation set (column no.)	NIVT	NOST	NOV	*mtry*	MSR	PVE	R^2^ training	RSE training	R^2^ validation	RSE validation	ASC of VS	RSE%
CV 1	1	11,127	553	40	3,709	0.499	81.9	0.976	0.256	0.800	0.787	1.675	46.967
CV 2	2	11,224	554	39	3,741	0.520	82.59	0.978	0.256	0.529	0.600	3.254	18.441
CV 3	3	11,060	562	31	3,686	0.534	80.29	0.976	0.257	0.962	0.386	1.460	26.451
CV 4	4	11,297	572	21	3,765	0.511	81.95	0.977	0.253	0.930	0.517	2.376	21.758
CV 5	5	11,249	572	21	3,749	0.505	82.59	0.977	0.257	0.601	0.739	3.410	21.680
CV 6	6	11,332	574	19	3,777	0.490	82.68	0.978	0.252	0.683	1.137	2.406	47.251
CV 7	7	11,315	576	17	3,771	0.506	82.42	0.977	0.257	0.650	0.742	3.648	20.344
CV 8	8	11,394	575	18	3,798	0.530	81.68	0.976	0.264	0.703	0.532	3.665	14.513
CV 9	9	11,300	576	17	3,766	0.504	82.44	0.978	0.253	0.142	1.087	3.800	28.605
CV 10	10	11,191	574	19	3,730	0.506	81.9	0.977	0.253	0.861	0.852	3.078	27.670
CV 11	11	11,162	561	32	3,720	0.509	82.83	0.978	0.256	0.305	0.739	3.223	22.925
CV 12	12	11,050	553	40	3,683	0.513	82.68	0.978	0.254	0.531	0.773	3.196	24.174
CV 13	13	11,190	561	32	3,730	0.522	81.22	0.977	0.254	0.852	0.799	2.367	33.745
CV 14	14	11,269	573	20	3,756	0.482	82.84	0.978	0.251	0.810	0.945	2.543	37.180
CV 15	15	10,971	555	38	3,657	0.533	80.91	0.975	0.263	0.938	0.445	1.900	23.398
CV 16	16	11,204	553	40	3,734	0.520	82.29	0.978	0.258	0.316	0.701	3.770	18.593
CV 17	17	11,356	561	32	3,785	0.483	83.28	0.978	0.250	0.471	0.844	3.737	22.589
CV 18	18	10,979	556	37	3,659	0.529	81.23	0.976	0.263	0.738	0.801	1.723	46.494
CV 19	19	11,061	553	40	3,687	0.505	81.51	0.977	0.251	0.858	0.797	2.190	36.406
CV 20	20	11,115	553	40	3,705	0.487	83.63	0.979	0.252	0.270	0.877	3.232	27.124

a Training set for all 20 random forest models is the rest of the 19 columns. NOST, number of samples in training set; NIVT, number of independent variables in training set; NOV, number of observations in validation set; MSR, mean of squared residuals; PVE, percentage variance explained; RSE, residual standard error; ASC, average sulfide concentration; VS, validation set.

To determine how well community metabolic structure predicts H_2_S concentration, a similar cross-validation was performed using feature-selected pathways (Table 5, Fig. S2). Results indicated that all the models were properly trained (mean percent variance explained = 79.179, SD = 0.815). Similar to the previous cross-validation models (based on unique sequence abundance), predictive performances for the treated columns (mean R^2^ = 0.814, SD = 0.077) were better than those for the nontreated columns (mean R^2^ = 0.421, SD = 0.224). The pathway-based RF models also displayed higher RSE % (*t *=* *5.025, *P = *0.0002) for treated columns (mean RSE % = 39.197, SD = 9.534) than for the nontreated columns (mean RSE % = 22.332, SD = 4.665). Comparison of both the cross-validation setups (based on community structure and metabolic profile) indicated that predictive performance of RF models based on community structure (mean R^2^ = 0.647, SD = 0.245) and that of RF models based on metabolic profile (mean R^2^ = 0.617, SD = 0.260) were not significantly different (*t *=* *0.376, *P = *0.7092).

**TABLE 5:** Details of cross-validation models for predicting sulfide concentrations based on relative abundance of feature-selected pathways^*a*^

Random forest model	Validation set (column no.)	NIVT	NOST	NOV	*mtry*	MSR	PVE	R^2^ training	RSE training	R^2^ validation	RSE validation	ASC of VS	RSE%
PVS 1	1	33	553	40	11	0.571	79.29	0.971	0.283	0.715	0.939	1.675	56.072
PVS 2	2	33	554	39	11	0.598	79.98	0.972	0.291	0.313	0.725	3.254	22.267
PVS 3	3	33	562	31	11	0.578	78.68	0.971	0.280	0.908	0.603	1.460	41.290
PVS 4	4	33	572	21	11	0.578	79.55	0.971	0.287	0.871	0.703	2.376	29.578
PVS 5	5	33	572	21	11	0.571	80.3	0.972	0.284	0.675	0.666	3.410	19.543
PVS 6	6	33	574	19	11	0.566	79.99	0.973	0.278	0.727	1.055	2.406	43.843
PVS 7	7	33	576	17	11	0.579	79.87	0.972	0.283	0.738	0.642	3.648	17.609
PVS 8	8	33	575	18	11	0.578	80.02	0.973	0.282	0.634	0.591	3.665	16.117
PVS 9	9	33	576	17	11	0.570	80.16	0.973	0.279	0.207	1.045	3.800	27.500
PVS 10	10	33	574	19	11	0.592	78.82	0.970	0.291	0.885	0.776	3.078	25.211
PVS 11	11	33	561	32	11	0.567	80.86	0.973	0.284	0.055	0.862	3.223	26.732
PVS 12	12	33	553	40	11	0.595	79.91	0.972	0.290	0.517	0.784	3.196	24.540
PVS 13	13	33	561	32	11	0.578	79.19	0.971	0.283	0.799	0.930	2.367	39.292
PVS 14	14	33	573	20	11	0.568	79.77	0.973	0.277	0.727	1.133	2.543	44.562
PVS 15	15	33	555	38	11	0.589	78.89	0.972	0.281	0.778	0.838	1.900	44.117
PVS 16	16	33	553	40	11	0.570	80.57	0.974	0.279	0.494	0.603	3.770	15.993
PVS 17	17	33	561	32	11	0.563	80.51	0.973	0.278	0.312	0.963	3.737	25.765
PVS 18	18	33	556	37	11	0.610	78.34	0.971	0.289	0.915	0.457	1.723	26.508
PVS 19	19	33	553	40	11	0.589	78.4	0.970	0.287	0.816	0.909	2.190	41.498
PVS 20	20	33	553	40	11	0.557	81.28	0.975	0.275	0.264	0.881	3.232	27.251

a Training set for all 20 random forest models is the rest of the 19 columns. NOST, number of samples in training set; NIVT, number of independent variables in training set; MSR, mean of squared residuals; PVE, percentage variance explained; RSE, residual standard error; ASC, average sulfide concentration; VS, validation set; NOV, number of observations in validation set.

### Prediction of microbial community source based on microbial community structure.

Prediction of microbial community sources can be important to evaluate for some systems since it is often hard to access the biofilms/sessile communities in inaccessible locations. A classification-based RF model was used to predict the source of the observed microbial community. Sessile communities were harvested for 19 columns at the end of the experiment. Three sand samples (from the top, middle, and bottom sections) from each column and three effluent samples from three time points before harvesting were used in this model. The RF model (CM 4) based on the 114 observations (Table 1) had a very low OOB estimate of error (0%) and high accuracies in predicting the sources (effluent or sessile) of the community (100% accuracies for prediction for both training and validation data set) (Table S5).

**FIG 7:**
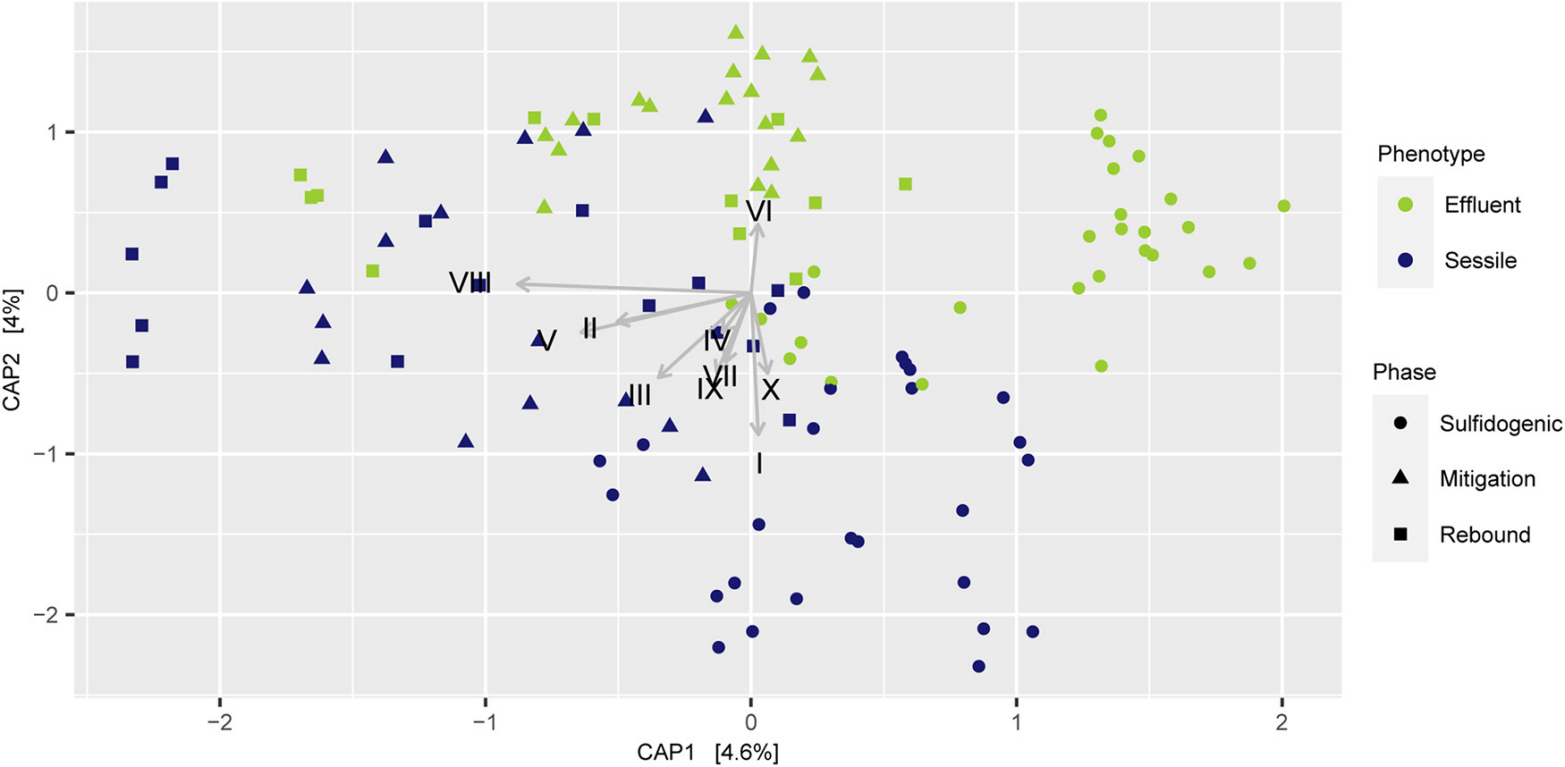
Canonical Analysis of Principal coordinates (CAP) of Bray-Curtis dissimilarity based on the relative abundance of unique sequences across 114 samples (used in model CM 4) and constraining the 10 most important taxa which are determinant for sessile-effluent classification. I, *Desulfoluna*, II, *Maribellus*, III, *Hyphomonas*, IV, *Alkalibacter*, V, *Rhodococcus*, VI, *Cupriavidus*, VII, *Lutibacter*, VIII, *Erythrobacter*, IX, *Halodesulfovibrio*, X, *Mangrovibacterium*. Detailed taxonomic affiliations are mentioned in Table 6.

The top 10 taxa critical for the prediction of sessile and effluent communities were selected based on the mean decrease in accuracy (Table 6). CAP analyses constraining the top 10 taxa displayed distinct clusters for effluent and sessile samples for most of the samples irrespective of the phases (Fig. 7). One (*Cupriavidus*) of the top 10 taxa was found to be higher in the effluent sample, whereas the rest of the 9 taxa among the top 10 were found to be higher in abundance in the sessile microbial community. It was interesting to note that the top five important taxa (*Desulfoluna*, *Maribellus*, *Hyphomonas*, *Alkalibacter*, *Rhodococcus*) were found to be higher in abundance in the sessile microbial community than in the effluent microbial community.

**TABLE 6:** Top 10 important taxa (based on unique sequence taxonomy) critical for the prediction of sessile-effluent communities^*a*^

CCG/CEG	ROPE taxonomy	MDA
*Desulfatibacillum aliphaticivorans*	*Desulfoluna*_genus_0.89	4.39
*Draconibacterium*	*Maribellus*_genus_0.83	4.28
*Hyphomonas*	*Hyphomonas*_genus_1.0	4.15
*Christensenella minuta*	*Alkalibacter*_genus_0.25	3.89
*Rhodococcus fascians* D188	*Rhodococcus*_genus_1.0	3.74
*Cupriavidus necator* N-1	*Cupriavidus*_genus_1.0	3.65
*Lutibacter profundi*	*Lutibacter*_genus_0.99	3.64
*Qipengyuania flava*	*Erythrobacter*_genus_1.0	3.63
*Desulfovibrionales*	*Halodesulfovibrio*_genus_1.0	3.35
*Labilibaculum antarcticum*	*Mangrovibacterium*_genus_0.48	3.32

a CCG, closest completed genome; CEG, closest estimated genome; MDA, mean decrease in accuracy; ROPE-based taxonomic affiliation is in the following format, taxon name_taxonomic rank_confidence of taxonomic affiliation.

## DISCUSSION

The application of machine learning (ML)-based models is a relatively new frontier in the field of microbial ecology. Though there are several studies where ML-based approaches were applied in the field of microbiology for the prediction of microbial species, diseases caused by microorganisms, interactions and associations among microorganisms, and environmental source of microorganisms (13–15), the application of ML for predicting biogeochemical processes is surprisingly limited (16, 17). Though many biogeochemical processes can be measured directly, transformations of compounds in a dynamic system often hinder the ability to measure the actual concentration of a product. This study described an approach that uses high-throughput phylogenetic placement and random forest models to predict H_2_S concentration from microbial community structures. This setup, which emphasizes the processes of sulfidogenesis and inhibition with nitrate salts, provides a good model system to evaluate the utility of machine learning for determining key environmental processes.

We first applied a PCoA analysis to identify major differences in community structure between phases. Although the PCoA plot (Fig. 3) represented the separation of the samples based on different phases, the samples from the sulfidogenic phase were found to be more widely distributed along axis 1. This suggests that different microbial communities can evolve in a system even if the environmental setting is similar and complicates the process of determining biogeochemical processes in a system from measurements of physicochemical parameters alone. Our results suggest that microbial community structure may be a more sensitive indicator of environmental transitions because it is more tightly coupled to biogeochemical processes than commonly measured physicochemical parameters. Despite reflecting many of the key dynamics, the PCoA was unable to separate the sessile and effluent communities. In contrast, the classification-based RF models were able to accurately predict the phases and sessile or effluent communities in the system. An additional key advantage over ordination is that RF can be used to develop a regression model to predict a continuous variable. Following the work of Thompson et al. (17), which accurately predicted DOC concentration from microbial community structure with RF, our RF models could predict H_2_S concentration with surprisingly high fidelity.

Variations in cell abundances across phases suggested that they can be an important tool for feature modification of microbial community data sets. However, RF regression models incorporating cell abundance did not perform significantly better than those based on relative abundance alone. Since the total cell abundances for each sample were distributed only among bacterial taxa and used for RF, abundance-based models could likely be improved by a better representation of archaeal community structure (limited here by the efficacy of the selected primer pair).

We have demonstrated that RF regression models can accurately predict biogeochemistry in a model system. This work is motivated by the need to predict biogeochemistry in field conditions, particularly for settings where the target geochemistry is very labile or highly transient in nature and thus difficult or impossible to observe directly. Under those conditions, the geochemistry may be reflected in the composition of the microbial community and successfully modeled from these data. This approach has several challenges, including the need to develop highly curated models for different geographical locations that may host taxonomically distinct communities. Because we expect the metabolic potential of the microbial community to be conserved more highly than the taxonomic structure, models based on predicted metabolic potential may be less sensitive to confounding factors such as geography or time. We made predictions of the metabolic structure for each bacterial community and used these data for the prediction of phases and H_2_S concentrations. This conversion of microbial community structure to metabolic profile is a form of feature engineering. Using this technique, we observed high accuracies for the prediction of phase and H_2_S concentration, suggesting that metabolic profiles are a reliable predictor of biogeochemical processes.

The proposed RF approach using pathway-based independent variables has an important limitation. Since the pathway abundances are predicted based on the closest estimated/completed genomes (24), strains not having a representative genome in the database may contribute to over-/underrepresentation of a specific pathway. Though this can be a limitation, the advantages of using pathway abundance models in comparison to the microbial community structure models are clear. In particular, the pathway-based models are expected to perform better across different sites than the microbial community structure models due to functional redundancy across different microbial communities associated with similar biogeochemistries. Moreover, the conversion of community structure into metabolic structure reduces the number of independent variables and generates parsimonious machine learning models. Improved performance of feature-selected pathway models further suggests that they are more parsimonious and may be more applicable to the real world. In this experiment, RF models also proved to be robust and generalizable based on the cross-validation experiment. Both the cross-validation setups (based on relative abundances of unique sequences and feature-selected pathways) had similar predictive performance, indicating that microbial community structures and metabolic profiles are equally reliable in predicting biogeochemical processes.

The RF-based models also have an advantage over ordination analyses, particularly when dealing with a huge number of independent variables. RF helps in selecting important features which are the main determinants of the dependent variables, at times providing us a new perspective on a dynamic system. In the present study, the closest completed genome (CCG) of the most important taxon for determining H_2_S concentration (Denitrovibrio acetiphilus DSM 12809) was found to be an NRB (25). Surprisingly, no known SRBs were observed among the 10 most important taxa for determining H_2_S concentration in this system. This indicates that nitrate reduction is a higher determinant factor for sulfidogenesis than sulfate reduction in a system where sulfidogenesis and mitigation (via nitrate addition) are taking place. This may result from the ubiquity of SRBs in the sulfidogenic and mitigation phases, in contrast to the much greater abundance of NRBs in the mitigation phase. The presence of NRBs is evidence of suppressed sulfidogenesis, but the presence of SRBs does not necessarily indicate enhanced sulfidogenesis since, under limiting conditions, SRBs can switch from sulfate reduction to fermentation or even nitrate reduction depending on their genomic repertoire (3, 9, 26).

Determining important variables can also be useful for determining sessile microbial community members since it is often hard to assess the biofilms/sessile communities in inaccessible locations. This study demonstrated the use of RF followed by constrained ordination in determining the microbial members of sessile and effluent communities. The sessile and effluent community members as suggested from this study can also be supported by their phenotypic traits. The presence of *Hyphomonas*, *Rhodococcus*, *Lutibacter*, and *Erythrobacter* (among the top 10 deterministic features for effluent/sessile prediction) in sessile communities in higher abundance can be supported by their biofilm-forming abilities (27–30), whereas the presence of Cupriavidus necator N-1 in higher abundance in the effluents can be supported by their cellular motility abilities (31).

The machine learning approach demonstrated in this paper can easily be applied to a microbial community data set for predicting the biogeochemical state of a system. This study also demonstrated that the conversion of microbial community structure into metabolic profiles could be used as a method for feature engineering in microbial ecology for predicting biogeochemical processes. In addition to predictive analytics, this study illustrates the applicability of random forest models in understanding the underlying microbial processes in a system. However, it is important to recognize that system-specific models will be needed for optimum predictive performance. Moreover, though it is possible to predict biogeochemical rates and standing stocks from microbial community structure, predicting biogeochemical state is a far easier task, particularly in dynamic systems with limited training data. We envision that ML-based models, along with high-throughput sequencing and analyses, will develop further as a valuable tool for determining biogeochemical processes and microbial ecosystem function in future microbiome research.

## MATERIALS AND METHODS

### Experimental setup.

As described in Dutta et al. (32), 20 upflow bioreactors (2.5 cm inside diameter 15 cm length, 74 cm^3^ volume, acrylic-jacketed glass columns) filled with ASTM graded sand, unground silica (U.S. Silica) were used to understand the shift in microbial diversity across different phases of sulfidogenesis and mitigation. Seawater was injected into all 20 bioreactors at a flow rate of 1 mL hr^−1^. Souring was initiated in all the columns under anoxic conditions. H_2_S concentrations were quantified in the system using the Cline assay (33). For H_2_S measurement, collection tubes were screwed onto the effluent line of the bioreactors for 1 h to collect samples, and the sample aliquot was withdrawn for further analysis. Volatile fatty acids were added to each column to promote sulfidogenesis and mitigation. A stock solution of 33 mM volatile fatty acids (VFAs; equimolar of acetate, butyrate, formate, and propionate) was fed to the influent flowline at a rate of 100 μL h^−1^ where it mixed with seawater flowing at 1 mL hr^−1^, leading to a column influent of 1.1 mL hr^−1^ with a total VFA concentration of 3 mM. Among 20 columns, 3.3 mM nitrate salts were applied to 10 columns (for mitigation of sulfidogenesis), whereas no nitrate treatment was involved in the remaining columns (Table S1). Four bioreactors (columns 7, 9, 10, and 14) were operated at ambient lab temperature (∼19°C), and the remaining 16 columns were operated at 30°C (Table S1).

Three main phases were observed in the treated columns, *viz*, sulfidogenic, mitigation, and rebound sulfidogenesis (referred to as rebound in this study). A transition to the mitigation phase (referred to as transition in this study) between sulfidogenesis and mitigation was determined where the H_2_S concentration was >1 mM even after the nitrate treatment. Small volumes (5 to 100 mL) of effluent samples were collected from each column over different time points and filtered through a Pall MicroFunnel filter funnel with 47 mm, 0.2 μm Supor filter. The filters were stored at −80°C until DNA extraction. The microbial diversities of the samples were determined to explore the shift in community structure across different time points and phases (Table S1). Nineteen columns were sacrificed at different time points, and the sessile communities from three different sections (top, middle, and bottom) of the columns were harvested under anaerobic conditions to understand the microbial diversity of the stationary phase (Table S1). The details of the experimental design are described in the supplemental material.

### DNA extraction, sequencing, and bioinformatics analysis.

For effluent samples, DNA was extracted from 674 filters (covering different columns across different time points) (Table S1) using the MagMAX microbiome ultra nucleic acid isolation kit, following the manufacturer’s protocol. Ninety-six-well standard plates were used for isolation of DNA using the KingFisher Flex bead handling robot. The MagMAX_Microbiome_Liquid_Buccal_Flex program provided by the manufacturer was used for DNA extraction. Columns were sacrificed and harvested during different phases (Table S1) to assess the sessile microbial community. Three sand samples (top, middle, and bottom) from each column (a total of 57 samples from 19 columns) were used for DNA extraction. For sand samples, the whole sections were harvested under anaerobic conditions and resuspended in DNA/RNA Shield (Zymo Research R1100-250) to preserve the samples and serve as a lysis buffer for homogenization. Subsequent processing for DNA extraction from sessile samples was conducted using ZymoBIOMICS DNA miniprep kit. A total of 731 samples (674 effluent samples and 57 sessile samples) were sequenced to an average depth of 40,709 paired-end reads (standard deviation [SD] = 10,527) on the Illumina MiSeq platform. Specifically, the V4 region of the 16S rRNA gene was PCR amplified with 515F-806R primers (34) that included sequencer adapter sequences used in the Illumina flowcell (35). Amplicon library preparations and sequencing were conducted at Argonne National Laboratory. The details of the sequencing are mentioned in the supplemental material. The sequence reads were submitted to the NCBI sequence read archive (SRA) under BioProject ID PRJNA714273 as reported previously (32).

Reads generated from the Illumina MiSeq were filtered, denoised, and merged using dada2 (36). The samples were split across three runs, and each run was denoised separately, considering different error profiles for different runs. The merged reads were inflated to redundant fasta files using the in-house script deunique_dada2.py (https://github.com/bowmanlab/seq_data_scripts/blob/master/deunique_dada2.py) for analysis with paprica. The output from deunique_dada2.py (*.exp.fasta) was analyzed using paprica v0.7.0 (https://github.com/bowmanjeffs/paprica/releases/tag/paprica_v0.7.0) for the determination of bacterial community and predicted metabolic structure (24). In brief, paprica placed each read on a phylogenetic reference tree created from complete 16S and 23S rRNA genes from all completed genomes in GenBank. Placements to terminal branches on the reference tree are referred to as CCG, while placements to internal branches are referred to as closest estimated genomes (CEG). The output of the paprica metabolic inference is an estimate of the enzymes and metabolic pathways contained in each member of the community. Further analyses were carried out using 16S rRNA gene copy number corrected abundances of unique sequences (can also be referred as amplicon sequence variant) generated using paprica. The paprica pipeline depends on RAxML-ng for reference tree construction (37) and Infernal (38) and EPA-ng (39) for phylogenetic placement. It further makes use of gappa (40) and pathway tools (41). The taxonomic affiliations of the unique sequences were confirmed using ROPE (https://github.com/avishekdutta14/ROPE). The detailed pipeline for paprica and ROPE is mentioned in the supplemental material.

### Cell counts.

Flow cytometry analysis of 553 samples was performed to determine effluent cell abundance across different phases in all 20 columns. One milliliter of effluent samples was collected during the same time points for DNA extractions from all the columns for cell counting using a Guava easyCyte 11HT Benchtop flow cytometer. The samples were fixed with 25% glutaraldehyde to a final concentration of 0.25% and stored at −80°C for further analysis. Before analysis, the samples were prefiltered using 60-μm filters to remove any larger debris. A 200-μL aliquot of each sample was transferred to a 96-well plate, stained with SYBR green (Molecular Probes), and spiked with a known number of 123count eBeads (Fisher Scientific). Cell abundance was determined from green fluorescence (excitation 488 nm/emission 525 nm) versus forward scatter using custom R scripts (https://github.com/bowmanlab/flow_cytometry_scripts). For further analyses, outliers (18 observations) for cell abundances from each phase were determined using Tukey’s method (42) and removed from the data set. An observation was considered to be an outlier when its value was outside the range Q1 − 1.5 × (Q3 − Q1), Q3 + 1.5 × (Q3 − Q1), where Q1 and Q3 are the first and third quartiles, respectively. Cell abundances for different bacterial taxa were generated using relative abundance data for each bacterial unique sequence (U) and total cell count per milliliter of a sample using the following equation:

$$\mathrm{absolute abundance of U (per mL)}=\frac{\mathrm{relative \% abundance of U}\;}{100}\times \mathrm{total cell count per mL}$$

### Random forest model and statistical analysis.

All the statistical and random forest model analyses were carried out in R and R Studio (43). GAMs based on average H_2_S concentrations and average cell abundances were constructed using the mgcv package (44). PCoA of Bray-Curtis dissimilatory based on the relative abundance of unique sequences across different samples was performed using phyloseq (45) to understand the shift in microbial diversity across time and phase. Random forests (RF) (46) classification and regression models were created using the randomForest package (47). Accuracies for classification models were determined using the confusionMatrix function from the caret package in R (48). For regression-based RF models, actual H_2_S concentration versus predicted H_2_S concentrations was plotted, and the R linear model function (*lm*) was used to determine the accuracies (from R^2^) and residual standard error (RSE) for the predictions. For all the models, 300 trees were generated, and the default *mtry* parameter (number of features randomly picked to split the tree at each node) was used for the classification and regression tasks, which is the square root of the number of features for classification and one-third of the number of features for regression. For both classification and regression models, 30% of the observations were randomly withheld for the validation and the remaining 70% were kept for training. For regression models, the variations in H_2_S concentrations in the validation data set and the training data set were kept similar to minimize the chance of underfitting the model. The codes used for RF classification and regression are present in https://github.com/avishekdutta14/Random_Forest.

For regression-based RF models, two time points and outliers based on H_2_S concentrations were removed. After filtering the data set based on inconsistencies and data availability, 609 effluent samples (out of 674 effluent samples) were left for further analyses. Outliers for H_2_S concentration from each phase were determined using Tukey’s method (42) and removed from the data set. An observation was considered to be an outlier when its value was outside the range Q1 − 1.5 × (Q3 − Q1), Q3 + 1.5 × (Q3 − Q1), where Q1 and Q3 are the first and third quartiles, respectively. After the outliers were removed, 593 effluent samples were present in the data set for further analysis. In order to minimize problems due to overfitting and to achieve parsimonious models, feature selection using the VSURF package (23) was used for pathway-based regression models. This package allows feature selection following three steps: step 1 eliminates irrelevant variables from the data set, step 2 selects variables related to the response, and step 3 refines the variable selection by eliminating redundancy in the set of variables selected in the second step for prediction purpose. Important variables were obtained from the random forest models based on percentage increase in mean squared error and mean decrease in accuracy for regression- and classification-based models, respectively. Canonical Analysis of Principal coordinates (CAP) of Bray-Curtis dissimilarity based on the relative abundance of the bacterial unique sequences across different samples and constraining the 10 most important taxa (determinant for H_2_S concentration/determinant for sessile-effluent classification) was performed using phyloseq and vegan packages (49). For the cross-validation experiment, RSE % was calculated using the following equation:

$$\mathrm{RSE \%=}\frac{\mathrm{RSE (calculated from linear models for validation data set)}}{\mathrm{mean of actual sulfide concentration of validation data set}}\times 100$$

Detailed description for random forest model construction is mentioned in the supplemental material.
