# Supplementary material for: Machine Learning Predicts Biogeochemistry from Microbial Community Structure in a Complex Model System
Source: Microbiol Spectr. 2022 Feb 9;10(1):e01909-21. doi: 10.1128/spectrum.01909-21 (PMC8826735; doi:10.1128/spectrum.01909-21)
Supplement: SUPPLEMENTAL FILE 1 — Supplemental material. Download SPECTRUM01909-21_Supp_1_seq11.pdf, PDF file, 0.5 MB [file spectrum01909-21_supp_1_seq11.pdf]

Supplementary Materials and Methods

Experimental setup

Twenty up-flow bioreactors were used to understand the shift in microbial diversity across different phases of biosouring and mitigation. *US Silica* ASTM Graded Sand – Unground Silica was used to pack the columns. Souring was first initiated using seawater and VFA flow-through, followed by subsequent flow-through and shut-in of yeast-extract-enriched seawater culture from anoxic serum bottles. Sulfide was measured in the system using the Cline assay (1). Seawater, collected from Scripps Pier, was injected into all the 20 bioreactors at a flow rate of 1 mL/hr. Volatile fatty acids (VFAs) were added in each column to promote sulfidogenesis and mitigation. 33 mM of VFAs (equimolar of acetate, butyrate, formate, and propionate) was added at a flow rate of 100 µL/h leading to a column influent concentration of 3 mM when mixed with 1 mL/hr seawater. The anoxic condition was initiated using a 100% N_2 flush to remove O_2, followed by a 100% CO_2 flush to remove gas bubbles during column commissioning. During experimentation, anoxic conditions were maintained by application of 99% N_2/1% CO_2 gas mixture sparged through the seawater reservoir. VFA preparations were also actively deoxygenated with N_2. Among 20 columns, nitrate salts (calcium nitrate salt in the form of Yara Petrocare 45) were applied to ten columns (for mitigation of biosouring), whereas no nitrate treatment was involved in the remaining columns (Table S1). This experimental setup was established at BP Biosciences Center.

There were three main phases that can be observed in the treated columns, *viz*: Sulfidogenic (S), Mitigation/Control (M), Rebound Sulfidogenesis (R). A transition to the mitigation phase (TM) between sulfidogenesis and mitigation was determined where the sulfide concentration was >1 mM even after the nitrate amendment. Four bioreactors (Columns 7, 9, 10, and 14) were operated under anaerobic conditions at ambient lab temperature (~19 °C), and the remaining 16 columns were operated at 30 °C (Table S1). The temperature was maintained by water circulation through column jacket assemblies. 30 °C was used to replicate conditions supporting mesophilic sulfate-reducing microorganism activity in oil reservoir bio-active zones. Effluent samples were collected from each column over different time points, and microbial diversity of the samples were determined to explore the shift in community structure across different time points and phases (Table S1). Nineteen columns were sacrificed at different time points, and the sessile communities

from three different sections (top, middle, and bottom) of the columns were harvested under anaerobic conditions to understand the microbial diversity of the stationary phase (Table S1).

Sequencing method

Three Illumina MiSeq runs were used to sequence 731 samples (674 effluent samples and 57 sessile samples). Briefly, PCR amplicon libraries targeting the 16S rRNA encoding gene present in metagenomic DNA were produced using a barcoded primer set adapted for the Illumina HiSeq2000 and MiSeq (2). DNA sequence data were then generated using Illumina paired-end sequencing at the Environmental Sample Preparation and Sequencing Facility (ESPSF) at Argonne National Laboratory. Specifically, the V4 region of the 16S rRNA gene was PCR amplified with 515F-806R primers (3) that included sequencer adapter sequences used in the Illumina flowcell (2). The forward amplification primer also contained a twelve-base barcode sequence that supports the pooling of up to 2,167 different samples in each lane (2, 4). Each 25 µL PCR reaction contained 9.5 µL of MO BIO PCR Water (Certified DNA-Free), 12.5 µL of QuantaBio's AccuStart II PCR ToughMix (2x concentration, 1x final), 1 µL Golay barcode tagged Forward Primer (5 µM concentration, 200 pM final), 1 µL Reverse Primer (5 µM concentration, 200 pM final), and 1 µL of template DNA. The conditions for PCR were as follows: 94 °C for 3 minutes to denature the DNA, with 35 cycles at 94 °C for 45 s, 50 °C for 60 s, and 72 °C for 90 s; with a final extension of 10 min at 72 °C to ensure complete amplification. Amplicons were then quantified using PicoGreen (Invitrogen) and a plate reader (Infinite® 200 PRO, Tecan). Once quantified, volumes of each of the products were pooled into a single tube so that each amplicon was represented in equimolar amounts. This pool was then cleaned up using AMPure XP Beads (Beckman Coulter) and then quantified using a fluorometer (Qubit, Invitrogen). After quantification, the molarity of the pool was determined and diluted down to 2 nM, denatured, and then diluted to a final concentration of 6.75 pM with a 10 % PhiX spike for sequencing on the Illumina MiSeq. Amplicons were sequenced on a 151bp × 12bp ×151bp MiSeq run using customized sequencing primers and procedures (2).

Bioinformatics analyses

731 paired-end reads generated from Illumina MiSeq were filtered, denoised, and merged using dada2 (5). Samples from three different runs were processed separately in dada2, considering different error profiles for different runs. The merged reads were inflated to redundant fasta files

using deunique_dada2.py (https://github.com/bowmanlab/seq_data_scripts/blob/master/deunique_dada2.py) for analysis using paprica. The output from deunique_dada2.py (.exp.fasta) was analyzed using paprica v0.7.0 (https://github.com/bowmanjeffs/paprica/releases/tag/paprica_v0.7.0) for the determination of community and predicted metabolic structure (6). In brief, paprica places each read on a phylogenetic reference tree created from complete 16S rRNA genes from all completed genomes in GenBank. Placements to terminal branches on the reference tree are referred to as closest completed genomes (CCG), while placements to internal branches are referred to as closest estimated genomes (CEG). The output of the paprica metabolic inference is an estimate of the enzymes and metabolic pathways contained in each member of the community. Further analyses were carried with 16S rRNA gene copy number corrected abundances generated using paprica. The taxonomic affiliations of the unique sequences were also determined using ROPE (https://github.com/avishekdutta14/ROPE). ROPE (RDP classification of paprica edges) determines the most abundant unique sequence (MAUS) affiliated to a particular taxonomic edge and uses RDP classifier to determine the taxonomic affiliation of MAUS. The taxonomic affiliation of the MAUS is then allocated to the affiliated edge. Another function of ROPE is to assign taxonomy to the unique sequences as obtained from paprica outputs.

Random Forest models

Random forests (RF) (7) classification and regression models were created using randomForest package (8) in the R statistical package to predict the phases and sulfide concentration. For classification-based RF models, relative and absolute percentage abundances of unique bacterial sequences were used as independent variables to predict different phases. Accuracies for such models were determined using confusionMatrix function from the caret package in R (9). For regression-based RF models, relative percentage abundances of unique bacterial sequences were used as independent variables to predict sulfide concentrations. Actual sulfide concentration vs. predicted sulfide concentrations was plotted, and linear model function (*lm*) in R statistical package was used to determine the accuracies (from R^2) and errors (residual standard error) for the predictions. For all the models, 300 trees were generated, and the default *mtry* parameter was used for classification, and regression task was used, which is the square root of the number of features

(for classification) or 1/3 of the number of features (for regression) randomly picked to split the tree at each node.

The out-of-bag error statistic provided in RF shows the goodness of model fit but not necessarily predictive performance. For this reason, 30% of the observations were randomly withheld and were used to perform more precise model validation. The data that was withheld for validation was termed as validation dataset, and the remaining dataset, which was used to train RF models was termed as the training dataset. Sulfide concentration variations in the validation dataset and training dataset were kept similar to remove the chances of underfitting.

Among 674 effluent samples, sulfide concentration was measured for 649 samples. For regression-based RF models, two time points (H and I) and outliers based on sulfide concentrations were removed. Samples from timepoints H (dated: 07/16/2019) and I (dated: 07/19/2019) were not considered for constructing regression-based RF models, since the sulfide concentrations for those timepoints were consistently low while compared to the sulfide concentrations from other S phase samples. Microbial diversity data along with other set of physicochemical parameters were studied for these data points, and it was concluded that these consistent lower values of sulfide concentration compared to the neighboring data points might be due to sampling or analytical error. Outliers for sulfide concentration from each phase were determined using Tukey's method (10). An observation was considered to be an outlier when its value was outside the range: $[Q1 - 1.5 \times (Q3 - Q1), Q3 + 1.5 \times (Q3 - Q1)]$, where Q1 and Q3 are the first and third quartiles, respectively.

In order to minimize problems due to overfitting and achieve parsimonious models, the VSURF package (11) was used. This package allows feature selection following three steps: Step 1 eliminates irrelevant variables from the data set, Step 2 selects variables related to the response, and Step 3 refines the variable selection by eliminating redundancy in the set of variables selected in the second step for prediction purpose.

Important variables were obtained from the random forest models based on the percentage increase in mean squared error. Percentage increase in mean squared error measures the effect on the predictive power when the value of a specific original variable is randomly permuted (7). If the random permutation drastically changes the predicted value (as measured by the mean squared error), then the original variable is considered critical (12). Differential abundance of the important

variables across different samples was analyzed using Canonical analysis of Principal Coordinated (CAP).

A cross-validation experiment was conducted using randomForest package in which samples from a column were used as a validation set, whereas the observations from the remaining 19 columns were used as the training set. Since the microbial communities shifted differently in different columns, this design was made to analyze the robustness of the RF models and to predict the sulfide concentration of samples, which was not used to train the RF models. To compare all the RF models, RSE % was calculated using the following equation

$$RME\ \% = \frac{RSE\ (calculated\ from\ linear\ models\ for\ validation\ dataset)}{mean\ of\ actual\ sulfide\ concentration\ of\ validation\ dataset} \times 100$$

RF models were also used to determine the source of the microbial community. The dataset for this model was prepared by including three sessile samples (from the top, middle, and bottom sections of the columns) and three effluent time points of each column (before the columns were sacrificed for sessile community harvesting).

References

1. Cline JD. 1969. Spectrophotometric determination of hydrogen sulfide in natural waters 1. Limnol Oceanogr 14:454–458.

2. Caporaso JG, Lauber CL, Walters WA, Berg-Lyons D, Huntley J, Fierer N, Owens SM, Betley J, Fraser L, Bauer M. 2012. Ultra-high-throughput microbial community analysis on the Illumina HiSeq and MiSeq platforms. ISME J 6:1621–1624.

3. Walters W, Hyde ER, Berg-Lyons D, Ackermann G, Humphrey G, Parada A, Gilbert JA, Jansson JK, Caporaso JG, Fuhrman JA. 2016. Improved bacterial 16S rRNA gene (V4 and V4-5) and fungal internal transcribed spacer marker gene primers for microbial community surveys. Msystems 1:e00009-15.

4. Caporaso JG, Lauber CL, Walters WA, Berg-Lyons D, Lozupone CA, Turnbaugh PJ, Fierer N, Knight R. 2011. Global patterns of 16S rRNA diversity at a depth of millions of sequences per sample. Proc Natl Acad Sci 108:4516–4522.

5. Callahan BJ, McMurdie PJ, Rosen MJ, Han AW, Johnson AJA, Holmes SP. 2016.

DADA2: high-resolution sample inference from Illumina amplicon data. Nat Methods 13:581.

6. Bowman J, Ducklow H. 2015. Microbial communities can be described by metabolic structure: A general framework and application to a seasonally variable, depth-stratified microbial community from the coastal West Antarctic Peninsula. PLoS One 10:e0135868.

7. Breiman L. 2001. Random forests. Mach Learn 45:5–32.

8. Liaw A, Wiener M. 2002. Classification and regression by randomForest. R news 2:18–22.

9. Kuhn M. 2008. Building predictive models in R using the caret package. J Stat Softw 28:1–26.

10. Tukey JW. 1977. Exploratory Data AnalysisAddison-Wesley, MA, USA.

11. Genuer R, Poggi J-M, Tuleau-Malot C. 2015. VSURF: an R package for variable selection using random forests.

12. Echeverry-Galvis MA, Peterson JK, Sulo-Caceres R. 2014. The social nestwork: Tree structure determines nest placement in kenyan weaverbird colonies. PLoS One 9:e88761.

Supplementary Tables

Column Name	Number of Samples (with sessile)	Number of Samples (without sessile)	Column Sacrifice stage	Treatment	Temperature (°C)
Column 1	47	44	Rebound	Treated	30
Column 2	47	44	Sulfidogenic	Not-treated	30
Column 3	38	35	Mitigation	Treated	30
Column 4	27	24	Mitigation	Treated	30
Column 5	27	24	Sulfidogenic	Not-treated	30
Column 6	25	22	Mitigation	Treated	30
Column 7	25	22	Sulfidogenic	Not-treated	19
Column 8	25	22	Sulfidogenic	Not-treated	30
Column 9	25	22	Sulfidogenic	Not-treated	19
Column 10	25	22	Mitigation	Treated	19
Column 11	38	35	Sulfidogenic	Not-treated	30
Column 12	47	44	Sulfidogenic	Not-treated	30
Column 13	38	35	Mitigation	Treated	30
Column 14	27	24	Mitigation	Treated	19
Column 15	47	44	Rebound	Treated	30
Column 16	47	44	Sulfidogenic	Not-treated	30
Column 17	38	35	Sulfidogenic	Not-treated	30
Column 18	47	44	Rebound	Treated	30
Column 19	47	44	Rebound	Treated	30
Column 20[#]	44	44	Not harvested	Not-treated	30

Table S1: Details of experimental design showing treated and non-treated columns

no sessile samples were harvested for Column 20

Table S2: Confusion matrix for validation dataset of model CM 1

	Reference			
Prediction	M	R	S	TM
M	45	2	0	0
R	0	15	0	0
S	0	0	131	11
TM	1	0	0	0

N.B.: M-Mitigation, R-Rebound sulfidogenesis, S-Sulfidogenesis, TM-Transition to Mitigation

Table S3: Confusion matrix for validation dataset of model CM 2

	Reference			
Prediction	M	R	S	TM
M	34	2	0	0
R	0	7	0	0
S	1	0	99	9
TM	0	0	0	0

N.B.: M-Mitigation, R-Rebound sulfidogenesis, S-Sulfidogenesis, TM-Transition to Mitigation

Table S4: Confusion matrix for validation dataset of model CM 3

	Reference			
Prediction	M	R	S	TM
M	46	1	1	1
R	0	14	0	0
S	0	2	129	10
TM	0	0	1	0

N.B.: M-Mitigation, R-Rebound sulfidogenesis, S-Sulfidogenesis, TM-Transition to Mitigation

Table S5: Confusion matrix for validation dataset of model CM 4

	Reference	
Prediction	Sessile	Effluent
Sessile	17	0
Effluent	0	17

Supplementary Figures

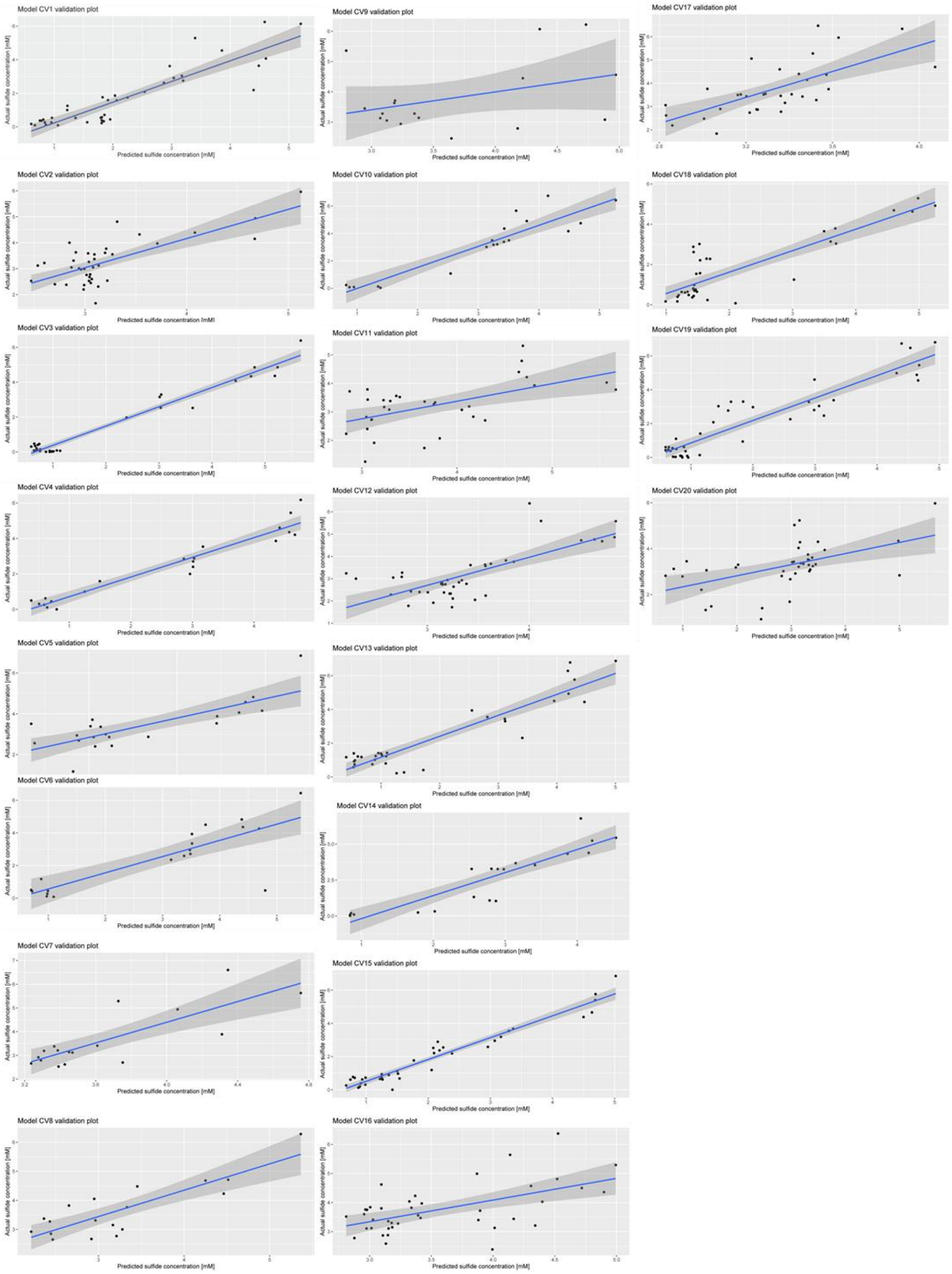

Figure S1: H_2S concentration prediction with cross-validation Random Forest models based on relative taxa abundance. Scatter plot of predicted versus actual H_2S concentration from validation set of all the models descried in Table 4.

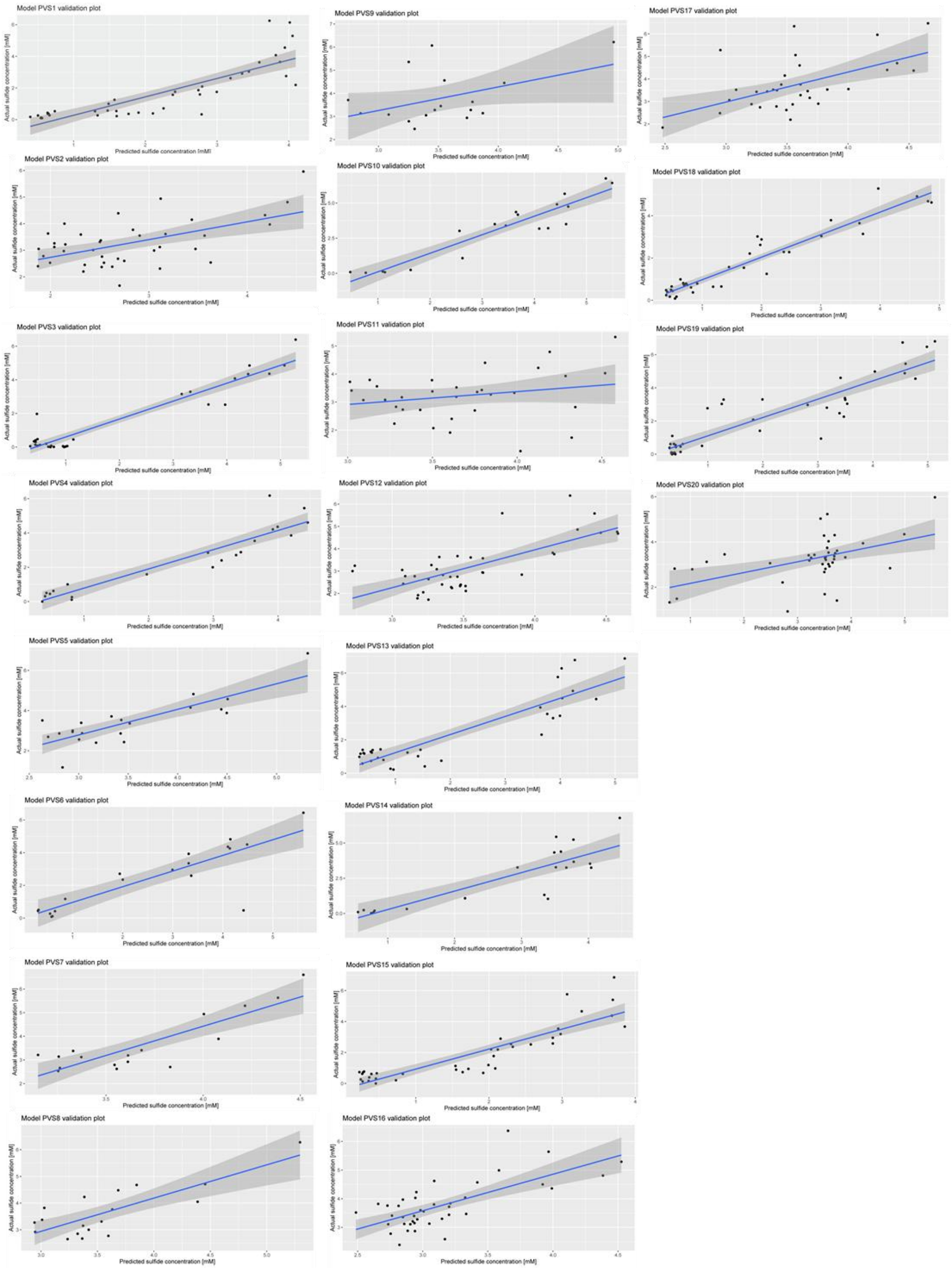

Figure S2: H_2S concentration prediction with cross-validation Random Forest models based on feature selected pathway abundance. Scatter plot of predicted versus actual H_2S concentration from validation set of all the models descried in Table 5.
